# Psychological adaptation and resilience among elderly refugees: protective and risk factors in confronting trauma and social isolation

**DOI:** 10.3389/fpsyg.2025.1725273

**Published:** 2025-12-10

**Authors:** Walaa Badawy Mohamed Badawy, Fatimah Salem Mohammed Alshehri, Khozam Saeed Ali Dakhil Allah, Hanan Ayidh saeed Alahmari

**Affiliations:** 1 Department of Psychology, College of Arts, Menoufia University, Shebeen El-Kom, Egypt; 2 Department of Psychology, College of Education, King Khalid University, Abha, Saudi Arabia

**Keywords:** elderly refugees, psychological adaptation, resilience, trauma, social isolation, protective factors, risk factors, mental health

## Abstract

**Background:**

Elderly refugees represent a profoundly vulnerable subgroup, facing the dual challenge of aging and the psychological sequelae of forced displacement. This study explores the psychological adaptation and resilience of elderly refugees, identifying key protective and risk factors in their confrontation with trauma and social isolation.

**Methods:**

A qualitative phenomenological design was employed, utilizing in-depth, semi-structured interviews with 13 elderly refugees (aged 60+) in Cairo, Egypt. Data were analyzed using Interpretative Phenomenological Analysis (IPA) to identify themes related to trauma, coping mechanisms, social support, and identity.

**Findings:**

The analysis revealed the enduring psychological impact of pre-migration trauma and post-migration isolation. Key protective factors fostering resilience included robust social support networks within the refugee community and the central role of religious faith as a coping mechanism. Primary risk factors exacerbating psychological distress were profound cultural bereavement, struggles with identity, and significant barriers to social integration. Participants articulated living “between two worlds,” highlighting the tension between preserving their heritage and adapting to the host society.

**Conclusion:**

The findings underscore the critical need for long-term, culturally sensitive mental health interventions tailored for elderly refugees. Psychological support services must integrate an understanding of these protective and risk factors. Mental health professionals, particularly psychologists, are pivotal in facilitating this support, advocating for policies that foster social inclusion, and implementing community-based programs that leverage existing resilience mechanisms to improve psychological outcomes for this population.

## Introduction

The global refugee crisis represents one of the most significant humanitarian challenges of our time, with millions forcibly displaced from their homes due to conflict, persecution, and environmental disasters. Within this vast displaced population, elderly refugees constitute a particularly vulnerable yet frequently overlooked subgroup. According to the United Nations High Commissioner for Refugees (UNHCR, 2023), individuals aged 60 and above account for a substantial portion of refugee populations, yet their specific needs and experiences remain largely marginalized in both policy and research agendas. This population faces the dual challenge of navigating the psychological sequelae of forced displacement while simultaneously coping with the physical, cognitive, and social changes associated with aging. The convergence of these factors creates a unique set of vulnerabilities that demand specialized attention from mental health professionals and researchers alike.

The existing literature on refugee mental health has predominantly focused on younger populations, particularly children, adolescents, and working-age adults (Porter and Haslam, 2005; Fazel et al., 2012). This focus is understandable given the long-term implications for human capital development and societal integration. However, this emphasis has created a significant knowledge gap regarding the psychological experiences of elderly refugees. While substantial evidence exists concerning the high prevalence of mental health disorders among refugee populations in general, including post-traumatic stress disorder (PTSD), depression, and anxiety (Blackmore et al., 2020), the manifestation, course, and treatment of these conditions in elderly refugees remain inadequately explored. The unique intersection of aging, trauma, and displacement represents a critical area requiring immediate scholarly attention.

Elderly refugees experience multiple compounded losses that profoundly impact their psychological well-being. These include not only the loss of homeland, material possessions, and social status but also the loss of loved ones, social networks, and cultural identity (Bhugra and Becker, 2005). The process of forced migration often severs connections to places of worship, community centers, and social gathering spots that have provided structure and meaning throughout their lives. Furthermore, many elderly refugees have experienced multiple traumatic events across their lifespan, including political violence, persecution, and dangerous migration journeys, creating complex trauma profiles that differ significantly from those of younger refugees (Nickerson et al., 2011). The cumulative effect of these losses and traumas, combined with age-related vulnerabilities, creates a psychological burden that existing mental health frameworks may not adequately address.

A comprehensive review of the literature reveals a substantial gap in research specifically addressing the psychological adaptation of elderly refugees. Hocking et al. (2021) conducted a systematic review of qualitative evidence on older refugees in humanitarian emergencies and found that “the voices and experiences of older refugees are largely absent from the qualitative literature on humanitarian emergencies” (p. 1). This neglect is particularly concerning given the demographic aging of global refugee populations. The existing research that does include elderly refugees often treats them as a homogeneous group, failing to account for the diversity of their experiences based on factors such as gender, socioeconomic background, country of origin, and specific trauma history (Hachem et al., 2022). This oversimplification limits our understanding of the nuanced ways in which different subgroups within the elderly refugee population experience and adapt to displacement.

The limited research that has focused on elderly refugees has primarily adopted a pathological perspective, emphasizing vulnerability, dependency, and mental health disorders rather than exploring factors that promote resilience and successful psychological adaptation (Siriwardhana et al., 2014). While understanding psychopathology is crucial, an exclusive focus on deficits provides an incomplete picture of the refugee experience. There is a pressing need for research that examines protective factors and resilience mechanisms that enable some elderly refugees to maintain psychological well-being despite profound adversity. As Southwick et al. (2014) argue, “studying how people overcome adversity may be as important as studying the origins and treatments of psychopathology” (p. 2). This balanced approach is particularly relevant for elderly refugees, who often demonstrate remarkable resilience despite multiple losses and traumas.

Methodologically, research on elderly refugees has been limited by several factors. First, many studies rely on quantitative measures developed for Western populations that may not adequately capture the cultural expressions of distress and resilience among elderly refugees from diverse backgrounds (Kohrt et al., 2014). The cross-cultural validity of standard psychological assessment tools for this population remains questionable, particularly when dealing with complex constructs such as trauma, grief, and resilience. Second, there is a scarcity of qualitative research that explores the subjective lived experiences of elderly refugees in sufficient depth. Phenomenological approaches that prioritize the voices and interpretations of elderly refugees themselves are notably absent from the literature (Smith et al., 2009). Without such nuanced understanding, mental health interventions risk being culturally insensitive and potentially ineffective.

Furthermore, existing research has often failed to situate the psychological experiences of elderly refugees within their broader social and cultural contexts. Mental health outcomes cannot be understood in isolation from factors such as social support networks, access to healthcare, legal status, experiences of discrimination, and the process of cultural adaptation (Miller and Rasco, 2004). An ecological perspective that considers the multiple systems influencing elderly refugees’ psychological adaptation is largely missing from the literature. This represents a significant gap, as understanding the interaction between individual, social, and structural factors is essential for developing comprehensive mental health support strategies for this population.

From a theoretical perspective, there is a notable gap in applying and adapting established psychological frameworks to understand the experiences of elderly refugees. Theories of acculturation (Berry, 1997), resilience (Southwick et al., 2014), and trauma (Herman, 1992) have been developed primarily based on studies with younger populations or non-refugee elderly individuals. How these theories apply to elderly refugees, who must navigate the dual challenges of acculturation and aging while coping with trauma, remains inadequately explored. For instance, Berry’s acculturation model identifies four strategies (integration, assimilation, separation, and marginalization) that individuals may adopt when navigating a new cultural context. However, how age-related factors influence the adoption and effectiveness of these strategies among elderly refugees represents an unexplored area of inquiry.

Similarly, existing models of resilience have primarily been developed and validated with Western populations, raising questions about their cross-cultural applicability. The specific protective factors that foster resilience in elderly refugees from diverse cultural backgrounds remain poorly understood. As Santiago et al. (2021) note, “there is a critical need to examine resilience from a cultural and contextual perspective, particularly among underserved populations such as refugees” (p. 15). Understanding the culturally specific manifestations of resilience in elderly refugees is essential for developing culturally sensitive mental health interventions.

Additionally, the role of mental health professionals in supporting the psychological adaptation of elderly refugees has received limited attention in the literature. While there is growing recognition of the importance of culturally competent care for refugee populations in general (Kirmayer et al., 2011), the specific competencies required to work effectively with elderly refugees have not been adequately delineated. This represents a significant practice gap that mirrors the research gap in this area. Mental health professionals working with this population must navigate complex issues related to trauma, grief, cultural adaptation, and age-related challenges, yet evidence-based guidelines for such work remain underdeveloped.

### The current study

This study aims to address these critical gaps in the literature by conducting a qualitative phenomenological investigation of the psychological adaptation and resilience of elderly refugees in Cairo, Egypt. Focusing specifically on the protective and risk factors that influence their mental health outcomes in the context of trauma and social isolation, this research seeks to center the voices and lived experiences of elderly refugees themselves. By adopting a phenomenological approach, the study prioritizes the subjective meanings that elderly refugees attribute to their experiences of displacement, trauma, and adaptation.

The study is guided by two primary research questions: (1) What are the protective factors that foster psychological adaptation and resilience among elderly refugees confronting trauma and social isolation? (2) What are the risk factors that exacerbate psychological distress and hinder adaptation in this population? In addressing these questions, this research aims to contribute to a more nuanced understanding of the psychological experiences of elderly refugees, with implications for the development of targeted, culturally sensitive mental health interventions. Furthermore, by identifying specific protective and risk factors, the study seeks to inform the work of mental health professionals, particularly psychologists, who are increasingly called upon to support this vulnerable yet resilient population.

## Methods

### Study design

A qualitative phenomenological investigation was conducted using Interpretative Phenomenological Analysis (IPA) as outlined by Smith et al. (2009). This methodological approach was selected for its capacity to provide detailed examinations of personal lived experience and its focus on how individuals make sense of their experiences. Given the complex psychological nature of trauma, adaptation, and resilience among elderly refugees, IPA offers an appropriate framework for understanding the subjective meanings that participants attribute to their experiences of displacement and aging in a foreign context.

The phenomenological orientation of this study aligns with the need to center the voices of elderly refugees themselves, whose perspectives have been historically marginalized in refugee research. This approach allows for an in-depth exploration of both the protective factors that foster psychological resilience and the risk factors that contribute to psychological distress in this population.

### Participants and sampling

The study consisted of a total of 13 elderly refugees aged 60 years and above residing in Cairo, Egypt. Participants were selected based on the following criteria:

#### Inclusion criteria

- Aged 60 years or older
- History of forced displacement from their country of origin
- Minimum of 5 years residence in Egypt
- Ability to provide informed consent
- Willingness to share experiences related to displacement and adaptation

#### Exclusion criteria

- Severe cognitive impairment affecting ability to participate in interviews
- Acute psychological distress that could be exacerbated by participation

The sample consisted of participants from diverse national origins including Syria, Sudan, and South Sudan, with an average length of displacement of 7.2 years. This diversity enabled the capture of varied cultural perspectives on psychological adaptation while maintaining focus on the shared experience of elderly forced migration.

### Data collection

Data collection occurred over a three-month period from June to August 2024. Semi-structured, in-depth interviews served as the primary data collection method, allowing for both consistency across participants and flexibility to explore individual experiences in depth.

#### Interview protocol development

The interview guide was developed through a comprehensive review of psychological literature on refugee mental health, resilience, and acculturation. The guide was pilot-tested with two elderly refugees not included in the final sample and refined based on their feedback. Key topics included:

1 Pre-migration trauma experiences

   •  “Can you describe the circumstances that led to your departure from your home country?”
   •  “What were the most psychologically difficult aspects of your displacement journey?”

2 Post-migration adaptation challenges

   •  “What psychological challenges have you faced in adapting to life in Egypt?”
   •  “How has the experience of aging in a foreign country affected your mental well-being?”

3 Protective factors and coping mechanisms

   •  “What personal strengths or resources have helped you cope with these challenges?”
   •  “How has your religious or spiritual beliefs influenced your ability to adapt?”

4 Social support systems

   •  “What role have social connections played in your psychological adaptation?”
   •  “How has your relationships with family, community, or other refugees supported your mental health?”

5 Identity and cultural continuity

   •  “How has your sense of identity changed since displacement?”
   •  “What aspects of your cultural heritage have been most important to preserve?”

#### Interview procedure

Interviews lasted between 60 and 90 min and were conducted in locations chosen by participants to ensure psychological comfort and safety. All interviews were audio-recorded with participant consent and supplemented by field notes documenting observational data and researcher reflections. The lead researcher, trained in trauma-informed interviewing techniques, conducted all interviews with sensitivity to potential re-traumatization.

### Data analysis

The data were analysed using Interpretative Phenomenological Analysis (IPA), which is particularly suited to exploring how individuals make sense of their personal and social worlds. The analysis process involved several systematic steps:

1. *Familiarization and immersion*: The researchers repeatedly read and re-read the interview transcripts to become thoroughly familiar with the data. This initial immersion involved listening to audio recordings multiple times, reading transcripts while noting initial observations, recording early impressions and reflections in a research journal, and developing an intimate understanding of each participant’s narrative.
2. *Initial noting and coding*: This stage involved detailed examination of each transcript through descriptive noting (recording explicit content), linguistic noting (analyzing language use), conceptual noting (exploring deeper psychological constructs), and psychological focusing (identifying emotional states and cognitive processes).
3. *Developing emergent themes*: Initial notes were transformed into concise psychological themes by clustering related codes, identifying patterns, formulating theme statements, and maintaining connection to original participant accounts.
4. *Searching for connections across themes*: The emergent themes were organized and integrated through mapping relationships, identifying super-ordinate themes, developing thematic networks, and creating visual representations of theme connections.
5. *Moving to the next case*: The entire process (steps 1–4) was repeated for each participant to maintain individual case focus and preserve unique psychological experiences.
6. *Looking for patterns across cases*: The final stage involved identifying shared psychological experiences, noting significant variations, developing a master list of group themes, and ensuring themes remained grounded in individual accounts.

### Rigor

The study adhered to the Consolidated Criteria for Reporting Qualitative Research (COREQ) to ensure rigor and trustworthiness through several strategic approaches:

- *Credibility*: The lead researcher conducted all interviews, ensuring consistency. Member checking was employed, and transcripts were reviewed by a second researcher to validate themes.
- *Dependability*: The research process was meticulously documented, creating an audit trail for transparency.
- *Confirmability*: Field notes and a reflective journal were maintained to document the researcher’s thoughts and decision-making processes, ensuring findings were based on participants’ experiences.
- *Transferability*: Detailed descriptions of the study setting, participants, and methodology were provided to allow readers to determine the applicability of the findings to other contexts.

### Ethical considerations

Ethical approval was obtained from the relevant institutional review board. All participants provided written informed consent after the study’s purpose and procedures were thoroughly explained. Confidentiality was maintained by anonymizing all data. Participants were informed of their right to withdraw at any time and were provided with information for accessing psychological support services if needed during or after the research process (see Table 1).

**Table 1:** Interview question script.

Main questions	Probing questions
“Can you describe your journey to this country and the challenges you faced?”	“What was the most difficult part of your displacement experience?” “How have these experiences affected your daily life?”
“How do you cope with the stress of being an elderly refugee in a new country?”	“What helps you get through difficult days?” “What makes it hard to adapt to your new environment?”
“What role do social relationships play in your life here?”	“Who do you turn to when you need help?” “How has your social network changed since displacement?”
“How has moving to a new country affected your sense of identity?”	“What aspects of your culture are important to maintain?” “How do you balance your original culture with the new one?”
“How would you describe your mental health since arriving here?”	“What affects your emotional well-being positively or negatively?” “How do you take care of your psychological health?”
“What role does faith or spirituality play in your life as a refugee?”	“How does your religious practice help you cope?” “Has your faith changed since displacement?”
“What has been your experience with healthcare services here?”	“What helps or hinders your access to psychological care?” “How do healthcare providers understand your needs?”
“What gives you hope for the future?”	“What are your main concerns about growing older here?” “What would improve your quality of life?”

### Findings

#### Characteristics of participants

The study included 13 elderly refugees with diverse demographic and background characteristics, as detailed in Table 2. The sample comprised 8 males (61.5%) and 5 females (38.5%), with ages ranging from 61 to 74 years (mean age = 67 years). Participants had been displaced for periods ranging from 5 to 10 years (mean duration = 7.2 years), indicating substantial experience with post-displacement adaptation challenges.

**Table 2:** Participants characteristics.

Participant ID	Gender	Age	Country of origin	Years since displacement	Health conditions	Education level
P1	Female	63	Sudan	7	Hypertension	Primary school
P2	Female	65	Syria	5	Diabetes	Secondary school
P3	Female	63	South Sudan	7	Arthritis	No formal education
P4	Female	67	Sudan	10	Hypertension	Primary school
P5	Male	74	Syria	5	Hypertension, diabetes	No formal education
P6	Female	63	South Sudan	7	Hypertension	Secondary school
P7	Male	61	South Sudan	9	Arthritis	Primary school
P8	Male	66	Sudan	8	Diabetes	No formal education
P9	Male	69	Sudan	10	Hypertension	Primary school
P10	Male	68	Syria	6	Hypertension	No formal education
P11	Male	72	Syria	5	Arthritis	Secondary school
P12	Male	65	Sudan	7	Diabetes	Primary school
P13	Male	71	Syria	10	Hypertension, arthritis	No formal education

All participants had experienced significant pre-migration trauma including loss of family members, exposure to violence, and hazardous displacement journeys. These experiences were compounded by post-migration challenges including language barriers, cultural adjustment difficulties, and limited access to appropriate healthcare services. Despite these challenges, participants demonstrated considerable resilience and adaptive capacity, utilizing various psychological and social resources to navigate their new environments.

The demographic characteristics of the sample reflect the diversity of elderly refugee populations in urban settings and provide rich insights into the varying experiences of psychological adaptation and resilience across different cultural backgrounds and personal circumstances.

### Thematic analysis

The analysis revealed four major themes concerning psychological adaptation and resilience among elderly refugees, as summarized in Table 3. These themes reflect the complex interplay between trauma, coping mechanisms, social support, and identity reconstruction in the context of forced displacement and aging.

**Table 3:** Thematic analysis of psychological adaptation.

Theme	Sub-themes	Description	Supporting quotes
1. Trauma and loss	1.1 Persistent trauma symptoms	Ongoing psychological impact of pre-migration trauma and displacement experiences.	*“I still have nightmares about the journey. I lost my brother and I see his face in my dreams every night.” (P3)*
	1.2 Complicated grief	Multiple losses including family, homeland, and social status.	*“I mourn not just for my son, but for my country, my home, everything I worked for my whole life.” (P5)*
2. Resilience strategies	2.1 Religious coping	Use of religious faith and practices as psychological resources.	*“My faith gives me strength. When I pray, I find peace that helps me endure the difficult times.” (P7)*
	2.2 Cognitive adaptation	Reframing experiences and finding meaning in adversity.	*“I tell myself this hardship has made me stronger. Everything happens for a reason in God’s plan.” (P9)*
3. Social support systems	3.1 Community Solidarity	Importance of ethnic and refugee community networks.	*“The Sudanese families here are my new family. We support each other like we are relatives.” (P4)*
	3.2 Family bonds	Role of family relationships in providing emotional support.	*“My children give me purpose to keep going. I must be strong for them despite my own pain.” (P2)*
4. Identity reconstruction	4.1 Cultural preservation	Efforts to maintain cultural identity and traditions.	*“I cook our traditional foods and speak our language at home. This keeps me connected to who I am.” (P6)*
	4.2 Acculturation challenges	Difficulties in adapting to new cultural environment.	*"I feel caught between two worlds - not fully here, not fully there. It’s confusing for someone my age.” (P10)*

#### Theme 1: trauma and loss

Participants consistently reported enduring psychological effects of their displacement experiences. The trauma manifested through persistent symptoms including nightmares, anxiety, and intrusive memories. As Participant 3 articulated: “The journey here was long and painful. I lost my brother on the way, and I still see his face every night in my dreams.” These psychological wounds were often reactivated by current stressors, creating a complex layering of past and present trauma.

The experience of multiple losses extended beyond personal bereavement to encompass cultural, social, and material dimensions. Participants grieved not only for deceased loved ones but also for their homeland, social status, and way of life. This complicated grief was compounded by the inability to perform traditional mourning rituals, leaving many with unresolved emotional pain.

#### Theme 2: resilience strategies

Despite significant psychological challenges, participants demonstrated remarkable resilience through various adaptive strategies. Religious faith emerged as a primary coping mechanism, providing both comfort and meaning. Participant 7 explained: “My faith is my anchor in this storm. It gives me the strength to endure suffering and maintain hope for better days.”

Cognitive adaptation strategies included positive reframing of experiences and finding purpose in adversity. Many participants described how their struggles had fostered personal growth and strengthened their character. This cognitive flexibility enabled them to maintain psychological equilibrium despite ongoing challenges.

#### Theme 3: social support systems

The critical role of social support emerged as a fundamental factor in psychological adaptation. Participants consistently emphasized how community networks within refugee populations served as vital protective factors against psychological distress.

##### Community solidarity

The shared experience of displacement created strong bonds among refugees from similar cultural backgrounds. Participant 4 described: “We have created our own small Sudan here. When one of us is sick, we all visit. When someone needs help with documents, we go together. This community has become our family.” These networks provided not only practical assistance but also emotional validation and a sense of belonging that counteracted feelings of isolation.

Observational data confirmed the existence of sophisticated mutual aid systems where elderly refugees exchanged resources, information, and emotional support. These informal networks often filled gaps in formal support systems, providing immediate assistance that governmental and international organizations were unable to offer.

##### Family bonds

Family relationships played a crucial role in maintaining psychological well-being, though these relationships were often strained by displacement. Participant 2 explained: “My children are my reason for surviving. When I feel hopeless, I look at them and remember I must be strong.” However, changing family dynamics and role reversals, where children often adapted more quickly than their elderly parents, created additional psychological stress.

The pressure to appear strong for younger family members sometimes prevented elderly refugees from expressing their own emotional needs, leading to emotional suppression and increased psychological burden. This was particularly evident in participants who described hiding their depression or anxiety to avoid worrying their families.

#### Theme 4: identity reconstruction

The process of rebuilding identity in a new cultural context emerged as a central challenge, with participants navigating complex negotiations between preserving their original cultural identity and adapting to their new environment.

##### Cultural preservation

Participants employed various strategies to maintain connection to their cultural heritage. Participant 6 described: “Every Friday, I cook the same dishes we ate at home. The smells, the tastes - they transport me back and remind me who I am.” These cultural practices served as psychological anchors, providing continuity and stability amidst disruptive life changes.

Religious and cultural rituals took on heightened significance in displacement, offering not only spiritual comfort but also opportunities for community gathering and cultural transmission. However, participants often faced practical barriers to maintaining these traditions, including limited access to traditional foods, appropriate spaces for gathering, and diminishing numbers of community members who remembered how to perform certain rituals.

##### Acculturation challenges

The process of cultural adaptation presented significant psychological challenges, particularly for elderly participants. Participant 10 articulated this struggle: “At my age, learning new ways is like trying to teach an old tree to bend in a new direction. I try to adapt, but it feels unnatural and exhausting.”

Language barriers emerged as particularly significant obstacles, limiting access to services, social interaction, and psychological support. Many participants described feelings of infantilization when they had to rely on others for translation, representing a painful loss of autonomy and social status.

The tension between preserving one’s original identity and adapting to the host culture created what several participants described as “living between two worlds.” This liminal state often resulted in feelings of not fully belonging to either culture, complicating the process of identity integration and psychological adaptation.

### Cross-cultural variations

The analysis revealed notable differences in psychological adaptation strategies across cultural groups. Syrian participants often emphasized education and professional background as core identity components, while Sudanese participants focused more on community and religious identities. These cultural variations highlight the importance of culturally-sensitive approaches to psychological support and the need to understand the specific meaning-making frameworks that different refugee groups bring to the adaptation process.

### Implications for psychological practice

The findings suggest several critical implications for mental health professionals working with elderly refugees:

- The need to incorporate cultural traditions and religious practices into therapeutic interventions.
- Importance of addressing multiple layers of loss and grief.
- Value of facilitating community connection and peer support.
- Necessity of acknowledging and working with acculturation stress.
- Importance of family-focused interventions that address intergenerational dynamics.

This comprehensive thematic analysis provides valuable insights into the psychological experiences of elderly refugees and offers important directions for developing effective, culturally-informed mental health interventions for this vulnerable population.

## Discussion

This study provides a nuanced understanding of the psychological adaptation and resilience processes among elderly refugees in Cairo, Egypt. The findings reveal a complex interplay between trauma experiences, resilience mechanisms, and cultural adaptation challenges that collectively shape mental health outcomes in this vulnerable population. Our results both corroborate and extend existing literature on refugee mental health while offering unique insights specific to elderly displaced populations.

The persistent trauma symptoms documented in our study align with previous research on the long-term psychological impact of forced displacement. Silove et al. (2017) emphasized that trauma symptoms often persist years after resettlement, particularly when compounded by ongoing stressors. Our findings extend this understanding by demonstrating how trauma manifests uniquely in elderly refugees, often intertwining with age-related vulnerabilities, cumulative life stresses, and complicated grief processes. The co-occurrence of pre-migration trauma with post-migration adaptation challenges creates a distinctive psychological burden that necessitates specialized therapeutic approaches tailored to this demographic.

The fundamental role of religious faith as a resilience mechanism substantiates earlier findings by Chen et al. (2020) regarding religious coping in refugee populations. Our research expands this understanding by revealing how elderly refugees utilize religious practices not merely for emotional comfort but as comprehensive frameworks for meaning-making, identity preservation, and social connection. This finding suggests that spiritually-integrated therapeutic approaches may be particularly effective for this demographic, supporting Pargament’s (2007) theory of religious coping as a multidimensional resource.

The critical importance of social support networks corroborates Miller and Rasco's (2004) ecological approach to refugee mental health. Our findings demonstrate that these networks serve multiple essential functions for elderly refugees: providing practical assistance, emotional validation, cultural continuity, and opportunities for reciprocal support that maintain personal dignity. The communal nature of coping observed in our study highlights the limitations of exclusively individual-focused interventions and underscores the necessity of community-based approaches that leverage existing social capital within refugee communities.

The complex challenges of identity reconstruction and acculturation reflect Berry (1997) acculturation theory while introducing crucial gerontological perspectives. Elderly refugees face distinctive challenges in balancing cultural preservation with adaptation, often experiencing significant generational differences in acculturation rates within their own families. This intergenerational dynamic represents an important consideration for family-focused interventions and supports Bhugra and Becker's (2005) concept of cultural bereavement as a significant psychological stressor.

Notably, our findings regarding the psychological impact of structural barriers and discrimination align with recent research by Hynie (2018) that highlights how post-migration stressors can significantly influence mental health outcomes. The daily experiences of marginalization and bureaucratic obstacles described by participants not only create practical challenges but also systematically undermine psychological adaptation by reinforcing feelings of otherness and powerlessness.

While our findings generally support existing literature, they also reveal certain distinctive patterns. The remarkable resilience demonstrated by participants despite multiple adversities challenges the predominant pathology-focused narrative in refugee mental health literature. This aligns with recent shifts toward strength-based approaches advocated by Panter-Brick (2014), who emphasizes the importance of recognizing resilience resources within refugee communities.

The study’s limitations must be acknowledged when interpreting these findings. The relatively small sample size, while adequate for qualitative research, limits generalizability. The cross-sectional design captures experiences at a single time point, whereas longitudinal research might reveal evolving adaptation processes. Additionally, the focus on urban refugees in Cairo may not reflect experiences in camp settings or other geographic contexts.

Despite these limitations, the study offers important implications for clinical practice, policy development, and future research. The findings strongly suggest that effective psychological support for elderly refugees must integrate multiple approaches: addressing trauma symptoms while simultaneously strengthening resilience resources, supporting cultural identity processes, and addressing structural barriers that impact mental health.

## Conclusion

This study illuminates the complex psychological landscape of elderly refugees, revealing both significant vulnerabilities and remarkable resilience capacities. The findings demonstrate that successful psychological adaptation involves dynamic processes of navigating trauma, leveraging social and spiritual resources, and reconstructing identity within new cultural contexts.

The research makes several important contributions to the field of refugee mental health. First, it addresses a significant gap in literature by focusing specifically on elderly refugees, a demographic often overlooked in both research and service provision. Second, it provides nuanced understanding of how resilience operates in this population, moving beyond pathology-focused perspectives to identify strengths and adaptive capacities. Third, it highlights the critical intersection of aging and displacement experiences, revealing unique psychological challenges and resources in this population.

The implications for clinical practice are substantial. Mental health professionals working with elderly refugees should:

- Develop trauma interventions that account for age-related vulnerabilities and cumulative stress.
- Integrate cultural and spiritual dimensions into therapeutic approaches.
- Implement community-based models that strengthen natural support networks.
- Address both psychological symptoms and structural barriers affecting mental health.

For policy development, the findings suggest the need for:

- Age-sensitive and culturally-appropriate mental health services.
- Support for community initiatives that enhance social connectivity.
- Professional training in geriatric refugee mental health.
- Integrated care models addressing both psychological and physical health needs.

Future research should build on these findings through longitudinal studies examining adaptation processes over time, investigation of cross-cultural variations in resilience manifestations, development of validated assessment tools for elderly refugee populations, and evaluation of integrated intervention models that address the multiple dimensions of psychological adaptation identified in this study.

In conclusion, supporting the psychological well-being of elderly refugees requires comprehensive, multi-level approaches that recognize their resilience while addressing their unique vulnerabilities. By building upon existing strengths and addressing systemic barriers, mental health professionals, policymakers, and researchers can collectively contribute to improving mental health outcomes for this resilient yet vulnerable population.

## Data Availability

The original contributions presented in the study are included in the article/supplementary material, further inquiries can be directed to the corresponding author.
